# The Effect of an Extremely Low-Frequency Electromagnetic Field on the Drought Sensitivity of Wheat Plants

**DOI:** 10.3390/plants12040826

**Published:** 2023-02-13

**Authors:** N. S. Mshenskaya, M. A. Grinberg, E. A. Kalyasova, V. A. Vodeneev, N. V. Ilin, N. N. Slyunyaev, E. A. Mareev, Y. V. Sinitsyna

**Affiliations:** 1 Department of Biochemistry and Biotechnology, N.I. Lobachevsky State University of Nizhny Novgorod, 603950 Nizhny Novgorod, Russia; 2 Institute of Applied Physics of Russian Academy of Sciences, 603600 Nizhny Novgorod, Russia

**Keywords:** extremely low-frequency magnetic field, Schumann resonance, drought, *Triticum aestivum* L.

## Abstract

Extremely low-frequency magnetic fields are thought to be capable of modulating the resistance of plants to adverse factors, particularly drought. Magnetic fields in this frequency range occur in nature in connection with so-called Schumann resonances, excited by lightning discharges in the Earth–ionosphere cavity. The aim of this work was to identify the influence of a magnetic field with a frequency of 14.3 Hz (which corresponds to the second Schumann harmonic) on the transpiration and photosynthesis of wheat plants under the influence of drought. The activity of photosynthesis processes, the crop water stress index, relative water content and leaf area were determined during drought intensification. At the end of the experiment, on the 12th day of drought, the length, and fresh and dry weight of wheat shoots were measured. The results obtained indicate a protective effect of the magnetic field on plants in unfavorable drought conditions; the magnetic field delayed the development of harmful changes in the transpiration and photosynthesis processes for several days. At the same time, in the absence of the stressor (drought), the effect of the electromagnetic field was not detected, except for a decrease in relative transpiration. In favorable conditions, there were only minimal modifications of the photosynthetic processes and transpiration by the magnetic field.

## 1. Introduction

Earth’s magnetic field (MF) is of great significance for the formation of the modern atmosphere and the evolution of life on Earth [1]. Along with the slowly varying geomagnetic field (the so-called main geomagnetic field), the biosphere is affected by alternating MFs of natural and man-made origin. Whereas the geomagnetic field does not change significantly during the life of an individual living organism, the intensity of variable MFs is permanently changing owing to various natural and man-made factors. This is the reason for the high interest of researchers in the effects of alternating MFs on living organisms, including plants [2,3]. Among the frequencies of alternating MFs in the super-low-frequency (SLF 30–300 Hz) and extremely low-frequency (ELF 3–30 Hz) bands, special attention is given to 50–60 Hz (a pronounced anthropogenic component) [4,5]. Less attention is paid to the frequencies of oscillations in the Earth–ionosphere resonator (7.8, 14.3, 20.8 Hz), which are called Schumann resonances (ScR) [6,7]. Despite the fact that the mechanisms of perception of variable MFs by plants are currently hypothetical, a number of works have reliably established the influences of MFs on the most important physiological processes, including photosynthesis, transpiration, respiration, cell division, antioxidant status, etc. [2,8,9,10]. The observed effects have been recorded mainly for alternating MFs of high intensity. In the case of alternating MFs of relatively low intensity, the effects are often weakly expressed or absent under stationary conditions [11,12], but they manifest themselves during transient processes when the environmental conditions change. In particular, in our previous works, such a pattern was established in relation to the activity of photosynthesis and the electric potential in wheat plants under the action of an alternating MF with a frequency of 14.3 Hz and an intensity of 3–180 μT, and the greatest effect was revealed under the influence of an ELF MF of 18 µT [13,14,15].

It is known that plant responses to changes in environmental conditions are formed with the participation of intracellular and intercellular signaling systems, the operation of which plays a decisive role in the development of plant resistance to stress factors [16,17]. The effects of MFs on plant resistance have been studied in relation to stress factors such as pathogens, salinization, cold stress, etc. [10,17,18,19,20,21]. Particular attention has been paid to the effects of MFs on drought tolerance owing to the importance of this stressor for agricultural production. The protective effects of low-frequency MFs in relation to various crops have been shown [18,20,21,22,23]. However, while the effects of MFs on plant resistance to drought have been established in the case of a preliminary treatment of seeds with MFs (seed priming), they have not been studied in relation to vegetating plants [21]. It should be noted that, like other abiotic factors, the same MF causes a more pronounced response when treating plants in the vegetative stage, rather than seeds [2,3,24,25,26]. In the case of seed priming, the influence of MFs is based on regulation at the genome level, which can provide long-term effects [27,28]. At the same time, in a case when an MF affects vegetating plants, the response may also be due to the direct influence of the MF on physiological processes, as well as on the signaling systems that control them.

The above assumption is based on the effects of ELF MFs on the activities of key signaling systems, including calcium, ROS, and the hormonal system, which have been identified to date [2,15,21,29,30,31]. In particular, the influence of an alternating MF with a frequency of 14.3 Hz on wheat plants has an effect on the activity of antioxidant enzymes—key participants in ROS signaling [32].

The purpose of this article is to analyze the effects of a magnetic field with a frequency of 14.3 Hz (close to the frequency of the second ScR harmonic) in combination with drought on the water status and photosynthetic activity of wheat plants. The main distinguishing features of our approach, which made it possible to obtain the results of this article, are as follows:

- Focus on studying the effect of a low-intensity alternating MF on plants under stress (drought);
- Focus on the Schumann range (from several Hz to several tens of Hz), particularly the second ScR harmonic (chosen on the basis of the results of our previous studies);
- The MF acting on plants throughout the entire growing period, particularly during the development of a response to the stressor;
- The treatment mode chosen based on the assumption that plant signaling systems play an important role in the responses to the MF and stress;
- The use of a wide range of diagnostic tools that enable the non-invasive monitoring of the activities of key physiological processes in plants during the entire period of observation.

## 2. Results

### 2.1. Electromagnetic Field Effects on Growth Parameters under Control and Drought Conditions

**Figure 1:**
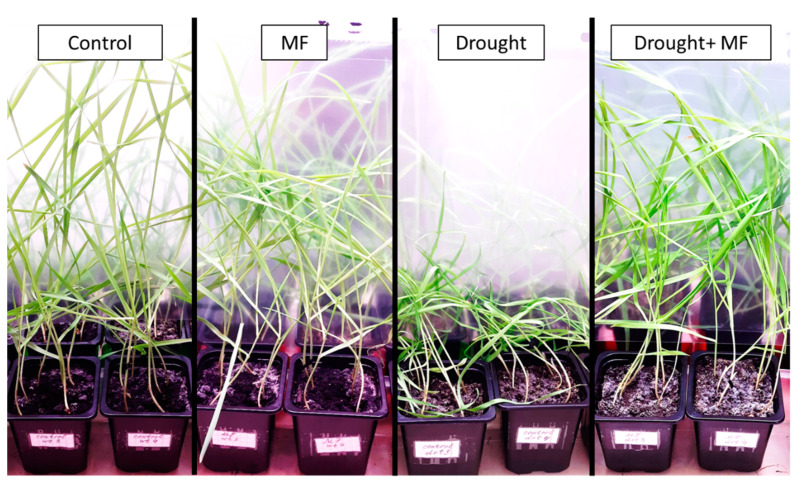
Photos of plants in various experimental groups (on the 12th day of drought).

The experimental magnetic field (frequency 14.3 Hz, magnetic induction 18 µT) did not cause significant changes in the appearance of the plants: there was no difference in appearance between the groups “Control” and “MF”. Only the plants exposed to drought (without MF) showed a loss of turgor (Figure 1).

**Figure 2:**
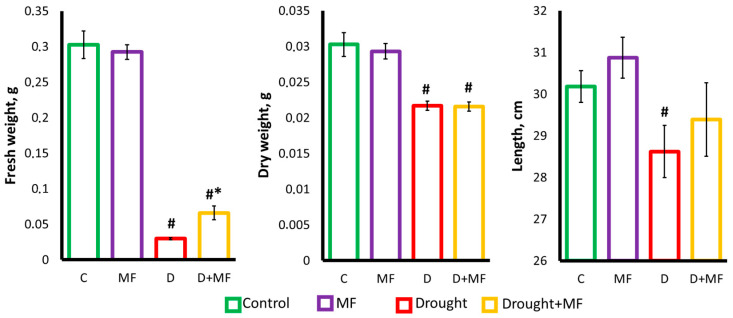
Fresh weight, dry weight and length of the wheat shoots grown under exposure to MF and drought. # indicates a significant difference compared to the “Control”, *p* < 0.05; * indicates a significant difference between “Drought + MF” and “Drought”, *p* < 0.05.

The experimental magnetic field did not affect the length or fresh and dry weight of wheat leaves in normal watering conditions (Figure 2). In the absence of the MF, a 12-day drought caused a loss of turgor in the plant shoots and a strong decrease in the fresh weight (by 10 times) and dry weight (by 27%). When plants growing under the action of the MF were exposed to drought, there was no such severe dehydration: the fresh weight of plants in the “Drought + MF” group was twice as much as that of the “Drought” group. At the same time, the dry weight and length of the plants in these two groups did not differ. Thus, the effects of the magnetic field on the mass and size of the plants did not appear in normal watering conditions but manifested under the influence of drought.

**Figure 3:**
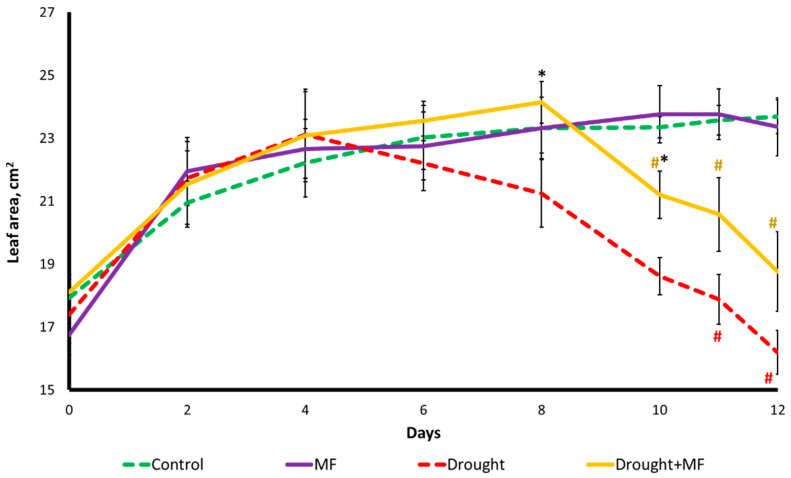
Dynamics of the leaf area of wheat plants grown under MF and drought conditions. The abscissa axis shows the days of drought after watering was discontinued. # indicates a significant difference compared to the “Control”, *p* < 0.05; * indicates a significant difference between “Drought + MF” and “Drought” *p* < 0.05.

The leaf area of wheat seedlings under normal watering conditions gradually increased. The magnetic field did not modify the leaf area of the “MF” group compared to the “Control” (Figure 3). During the drought, the visible leaf surface area decreased. Without the experimental magnetic field, the decrease began on the 6th day of the drought. For the plants grown in the magnetic field, the decrease in the visible leaf area started only on the 10th day of the drought (i.e., it was delayed by 4 days). The decrease in the visible leaf area during the drought is most likely due to the loss of turgor, since the fresh weight of the plants in the drought conditions greatly decreased, and the length of the leaves did not change significantly (Figure 1, Figure 2 and Figure 3).

### 2.2. Electromagnetic Field Effect on Photosynthesis under Control and Drought Conditions

**Figure 4:**
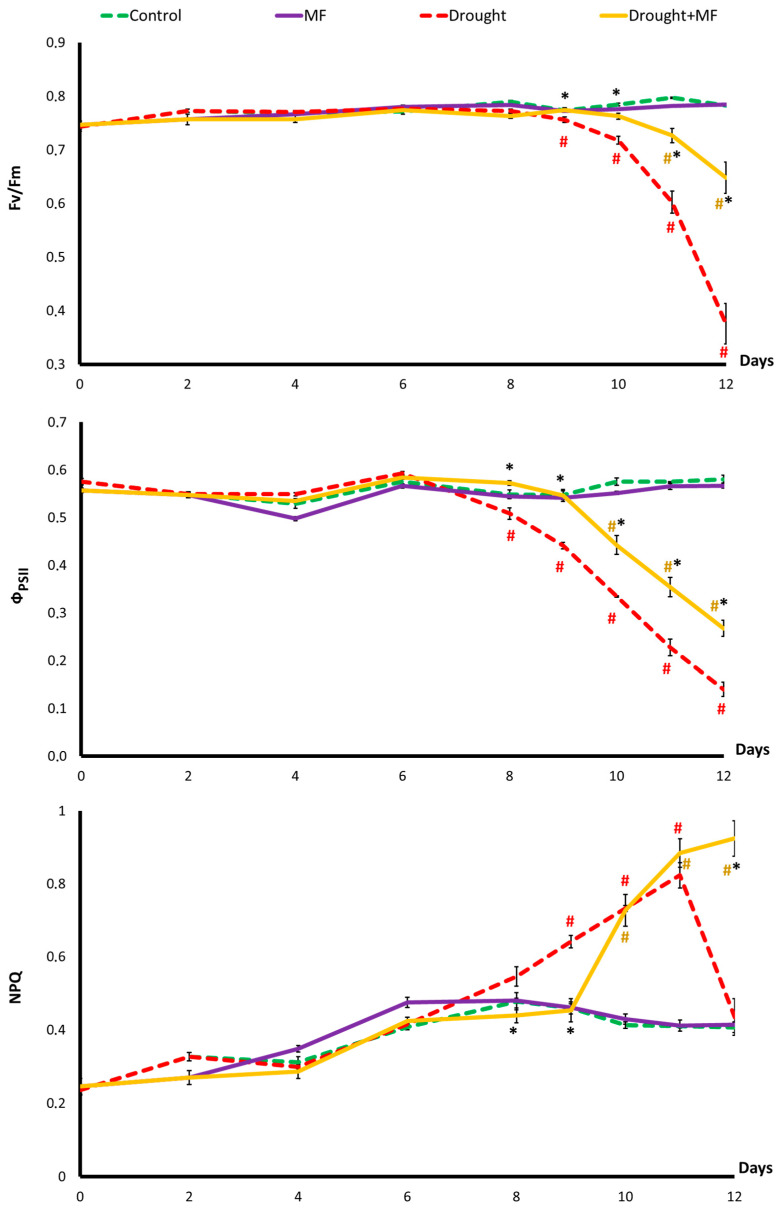
Photosynthesis activity indexes for the leaves of wheat plants grown under MF and drought conditions. The abscissa axis shows the days of drought after watering was discontinued. # indicates a significant difference compared to the “Control”, *p* < 0.05; * indicates a significant difference between “Drought + MF” and “Drought” *p* < 0.05.

With a normal water supply, the magnetic field did not affect the light-dependent photosynthesis reaction: the dynamics of Fv/Fm, Ф_PSII_, and NPQ in the “Control” and “MF” groups did not differ (Figure 4). In the absence of the MF, the drought modified all of the registered photosynthesis parameters. First, Ф_PSII_ decreased starting from the 8th day of the drought. Then, the NPQ increased sharply starting from the 9th day. Finally, on the 10th day after the start of the drought, a sharp decrease in the Fv/Fm began, which indicated the disintegrity of the photosynthetic apparatus. The magnetic field slowed down all the registered effects of the drought on the photosynthetic reactions. The changes in the “Drought + MF” group were similar to those in the “Drought” group but manifested themselves 1–2 days later. Thus, in our experiment, an MF with a frequency of 14.3 Hz had a protective effect on photosynthetic reactions in wheat leaves, expressed in the deceleration of the development of destructive processes in them.

### 2.3. Magnetic Field Effect on Water Status under Control and Drought Conditions

**Figure 5:**
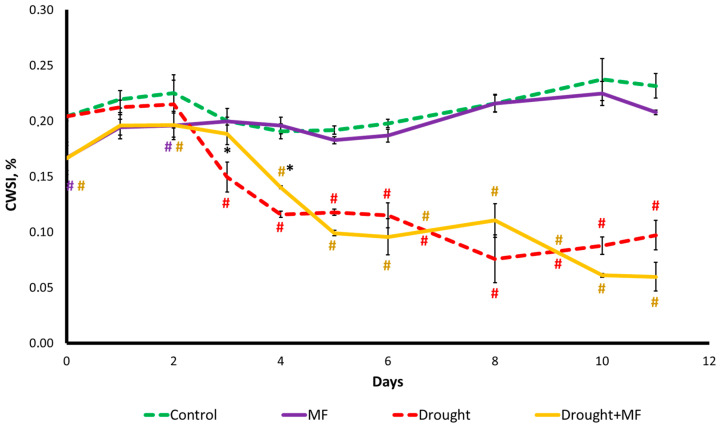
Coefficient of relative transpiration of wheat leaves grown under MF and drought conditions. The abscissa axis shows the days of drought after watering was discontinued. # indicates a significant difference compared to the “Control”, *p* < 0.05; * indicates a significant difference between “Drought + MF” and “Drought” *p* < 0.05.

In the case of regular watering, plants in the “MF” group had a crop water stress index (CWSI) 20% lower than those in the “Control” group (Figure 5). A lower level of transpiration was observed in the youngest plants, and from the third day of CSWI monitoring there was no difference between the plants in the “Control” and “MF” groups.

In the case of drought, on the 3rd day after the cessation of watering, the CWSI was 1.3 times lower than the control. On the 4th–6th days of the drought, it was two times lower, and on the 8th–11th days, it was three times lower than the control level. In the “Drought + MF” group, the CWSI also decreased, but with a delay of 1–2 days before the response. In general, the changes in transpiration in the “Drought” and “Drought + MF” groups were similar but differed in the time of onset of the decrease in their levels.

**Figure 6:**
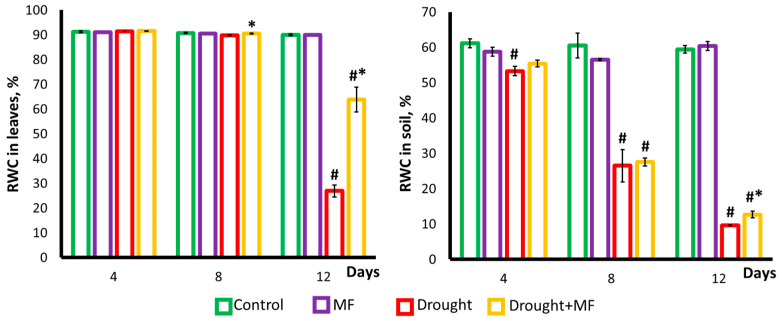
The relative water content (RWC) in the leaves of wheat plants and in the soil. The abscissa axis shows the days of drought after watering was discontinued. # indicates a significant difference compared to the “Control”; * indicates a significant difference between “Drought + MF” and “Drought”.

During regular watering, the MF did not affect the relative water content (RWC) in the plants and soil (Figure 6).

In the case of the drought, the magnetic field action was expressed in a significantly higher value of the RWC index of the leaves in the “Drought + MF” plant group (64%) compared to the “Drought” (27%) on the 12th day after watering was discontinued. At the same time, the MF contributed to the preservation of a higher level of soil RWC, namely, 13% in the “Drought + MF” group compared to 10% in the “Drought” group.

Thus, in normal watering conditions, there was no effect of the experimental MF on the growth, photosynthesis and water status of the wheat plantlets. A protective effect of the magnetic field on the plants was observed in unfavorable drought conditions: the magnetic field delayed the development of adverse changes for several days.

## 3. Discussion

The experiments showed that continual exposure to an MF with a frequency of 14.3 Hz (the second ScR harmonic) and a magnitude of 18 µT did not affect the morphometric parameters (shoot length, dry and fresh weight) of wheat plants in normal conditions (Figure 1). In the experiments of other authors using similar exposure parameters, either there was also no effect of the MF on the morphometric parameters, or there was a stimulation caused by the MF [2,9,10,18,19]. However, when analyzing and comparing the results, it should be taken into account that the existing literature, as a rule, concerns the short-term irradiation of seeds with MFs of high intensity and different frequencies, which differs from our exposure conditions. This may influence the results obtained for the following reasons: the effects of short-term, abrupt exposure and continual exposure differ significantly; the effect on seeds may differ from the effect on plants in the vegetative stage; the strength and direction of the response depend on the intensity of the factor, owing to the complex shape of the dose–response relationship; and the effects of the selected frequency (corresponding to the ScR), hypothetically, may differ from the effects of other frequencies [7,26].

Morphometric parameters of plants are largely determined by the activities of photosynthetic processes. In our experiments, the MF did not affect the main indicators of the light-dependent stage of photosynthesis (Fv/Fm, Φ_PSII_, NPQ) when plants were grown in the absence of the stressor (groups “Control” and “MF”) (Figure 4). This agrees with the data from the literature, according to which the activity of photosynthesis, as well as the morphometric parameters, does not change under the action of MFs with characteristics similar to ours [13,14,18]. At the same time, some studies have demonstrated the stimulating effect of the MF [2,8,9,10]. It has been shown that the MF influence is realized due to both changes in the number of structural components (photosynthetic pigments and individual photosynthetic enzymes, for example, Rubisco) and changes in the activity of the light-dependent reactions associated with the electron transport chain and changes in the rate of absorption of CO_2_ [2,8,10,13,14,19,33,34].

Despite the fact that, in the absence of the stressor, exposure to the MF does not affect integral indicators of the state of plants such as the morphometric parameters and photosynthesis activity, the effect of the MF on stomatal conductivity has been observed. In the presence of the MF, the stomatal conductivity, estimated by the value of the CSWI index, was at a lower level compared to the control during the growth of wheat seedlings (Figure 5). It is reported in the literature that the MF increases the water content in the body, reduces the magnitude of water and osmotic potentials, and, in general, “improves” water balance [8,10,19,20,35]. This effect can be based on both structural (an increase in the width and area of the veins or an increase in the amount of cuticular wax around the stomata) and functional changes (including a decrease in stomatal conductivity under the influence of the MF), as described in a number of works [18,27,36]. As a potential mechanism of the influence of the MF on stomatal conductivity, one can assume a change in the content of the phytohormone ABA, which controls the process of transpiration in plants. In turn, it should be emphasized that the pathways of ABA synthesis and signaling are closely related to ROS [37], and ROS signaling processes are now considered to be one of the main mechanisms of MF influence on plants [2]. In view of the lack of data in the literature on the effects of MFs on the hormonal system of plants, such an assumption requires additional experimental verification. 

As the most interesting result obtained in this work, it is necessary to note the effect of a low-frequency MF on wheat plants, which is weakly expressed under normal conditions but manifests itself far more strongly in the presence of an additional stress factor, namely drought. The MF had a pronounced protective effect, as indicated by the higher morphometric parameters of plants from the “Drought + MF” group compared to plants from the “Drought” group (Figure 1). The observed effect of the MF on plants during the vegetative stage is in good agreement with earlier findings indicating that the effect of MFs on seeds increases the resistance of adult plants to various unfavorable factors, including drought, salinization, excessive contents of heavy metals, cooling, damage by pathogens, etc. [10,18,19,20,38].

When analyzing the mechanisms of the protective effect of MFs in drought conditions, most attention was paid to the activity of photosynthesis owing to its close relationship with plant growth. During the experiments, it was shown that both in the “Drought” group and in the “Drought + MF” group, water deficiency caused a decrease in the morphometric parameters and an inhibition of photosynthesis, which was expressed first as a regulated decrease in activity (an increase in NPQ and a decrease in Φ_PSII_) and then as a violation of the structural integrity of the photosynthetic apparatus (a decrease in Fv/Fm) (Figure 3 and Figure 4). It was found that the protective effect of the MF observed in our experiments is expressed mainly in a shift of the time of onset of drought-induced responses, rather than in a change in their magnitude. This pattern was shown for both the morphometry and estimated by leaf area and for all parameters of the activity of the photosynthetic apparatus.

The intensity of photosynthesis in drought conditions may decrease owing to (1) the lack of CO_2_ due to the closure of the stomata and (2) disturbances in the activity of individual photosynthetic processes, followed by the occurrence of structural damage caused by the lack of water in the tissues [39,40,41,42]. The closing of stomata to conserve water and the opening of stomata to maintain CO_2_ concentration in drought conditions are competing processes [42]. Apparently, in our experiments, the decrease in photosynthesis was mainly a consequence of the decrease in the water content in the tissues and not of a CO_2_ deficiency caused by the closure of stomata. This is indicated by the parameters of the dynamics of these characteristics. Our experiments showed that in drought conditions, stomatal conductivity rapidly decreases (Figure 5), but the decrease in photosynthesis activity (Figure 4) begins only a few days afterwards. At the same time, the beginning of the decrease in photosynthesis coincides with the beginning of the decrease in the water content (Figure 6). It should be noted that, despite the difference in dynamics, stomatal conductivity and water content in tissues are closely related. The water content in the tissues of wheat plants remains at a high level for a long time, even at the onset of water deficiency in the soil (Figure 6). This is apparently associated with the early decrease in the conductivity of the stomata (Figure 5). Since the decrease in stomatal conductivity is one of the earliest responses to drought, one can assume that it is initiated by the propagation of some highly sensitive remote stress signals from the root that can cause the stomata to close [41,43]. The MF shifted the time of the beginning of the decrease in stomatal conductivity and then that of the water content of plants in the “Drought + MF” group (Figure 5 and Figure 6). This result correlates well with the effects of the MF on the dynamics of photosynthetic and morphometric parameters during drought.

The most likely reason for the longer maintenance of water in plants in the case of the increased MF is the initially different level of stomatal conductivity (Figure 5). The lower level of transpiration in plants exposed to the MF could contribute to a slower loss of water from the soil during the onset of drought as compared to the control. This may underlie the shift in the onset of the response to drought for all the other studied parameters.

In view of the known effects of MFs on the ROS content and activity of the antioxidant system [2,21,29,31], it can be assumed that the protective effect of the MF during drought may also be partly due to a modification of the redox balance maintenance system. In our earlier work, we showed the effect of an MF with identical parameters on the activity of antioxidant enzymes in wheat [32]. Considering that the damage during drought is closely associated with an increased level of ROS, this assumption seems reasonable but requires further analysis.

In general, the obtained results indicate that the effect of the low-frequency MF on wheat plants is much more pronounced in the presence of an additional factor than without the stressor. The observed effect is consistent with the earlier reported effects of MFs with similar characteristics on the activity of photosynthesis and the magnitude of the electric potential, which were also more pronounced in “transitional” (during the transition from darkness to light), rather than stationary, conditions [13,14,15].

## 4. Materials and Methods

### 4.1. Experiment Design

**Figure 7:**
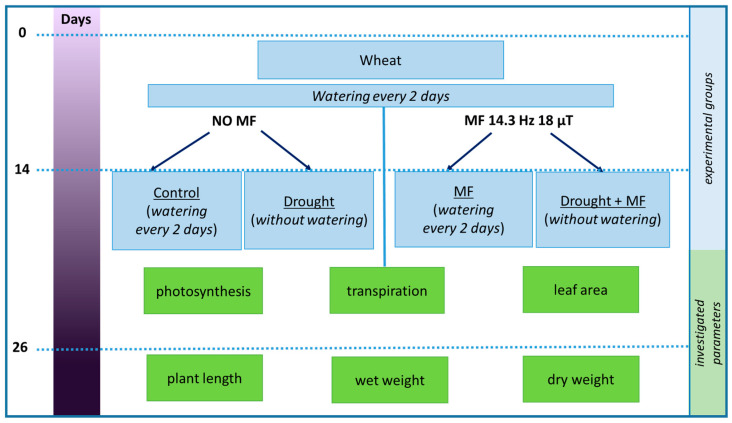
Scheme of the experiment.

The objects of the study were wheat plantlets (*Triticum aestivum* L.). The plants were sprouted and grown for 14 days and then divided into two groups: one group (NO MF) was in a geomagnetic field, while the other (MF) grew when an electromagnetic field was applied with a frequency of 14.3 Hz and magnetic induction of 18 µT (Figure 7). During this time, the watering of all the plants was regular—once every 2 days. 

**Figure 8:**
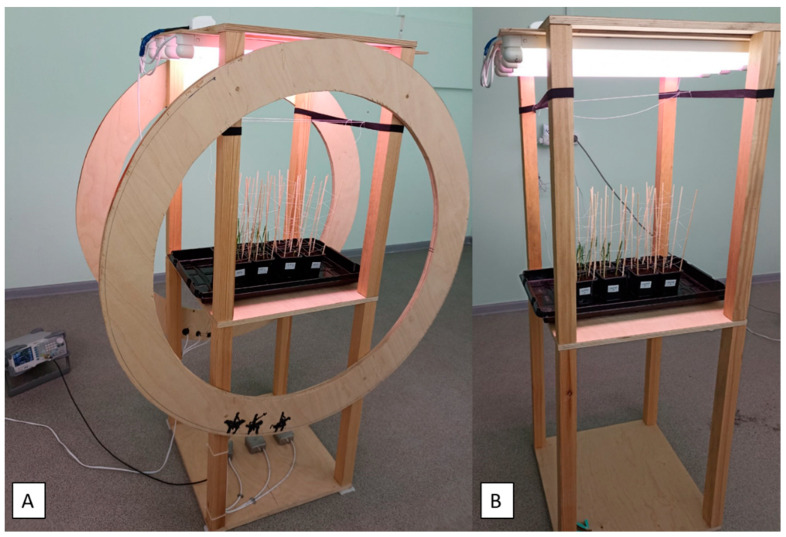
The experimental system and the position of the wheat plants in the Helmholtz coils (**A**) and without ELF MF (**B**).

The experimental alternating magnetic field was created using coaxially arranged Helmholtz coils mounted on a wooden device with lighting. A region of a homogeneous magnetic field with a diameter of 20 cm was located in the center between the coils (Figure 8A). The given value of the magnetic field amplitude was a calculated value based on the geometry and current of the coils. The presence (absence) of the magnetic field was monitored with a simple inductive sensor. The control samples were based on a similar design, but without Helmholtz coils (Figure 8B).

After two weeks, each of the groups was divided into 2 parts (Control and Drought). The control plants continued to be watered in the same mode. In the drought groups, watering was discontinued completely. 

After the watering of the plants was discontinued, the activity of photochemical processes, leaf area and the transpiration coefficient were registered. On days 4, 8 and 12 of drought, the relative water content in the plant leaves and in the soil was determined. At the end of the experiment, on the 12th day of drought, the length and fresh and dry weight of the wheat shoots were measured. 

### 4.2. Measurement of Growth Parameters 

The length of the wheat plants was measured on the second leaf. The fresh and dry weights of the plantlets were registered. For the dry weight estimation, the plants were dried for 4 h at 85 °C to a constant weight. 

The total area of plant leaves from one vegetative vessel was determined by chlorophyll fluorescence using the Data Analysis Software Version 5.6.7-64b for Plant Explorer^Pro+^ (PhenoVation, Wageningen, The Netherlands). 

### 4.3. Measurement of Photosynthesis and Leaf Area 

The photosynthesis activity was measured using the PAM imaging system Plant Explorer^Pro+^ (PhenoVation, Netherlands). Seedlings were adapted to dark conditions for 20 min. The first saturation pulse (SP) with an intensity of 2881 μmol m^−2^s^−1^ was used for the estimation of the initial and maximum rates of photosystem II fluorescence (F0 and Fm, respectively). Actinic light (AL) with an intensity of 136 μmol m^−2^s^−1^ was used on the first SP. Parameters of photosystem II, including the potential quantum yield of photosystem II (Fv/Fm), the effective quantum yield of photosystem II (Ф_PSII_) and the non-photochemical quenching of the chlorophyll fluorescence (NPQ), were calculated on the basis of F0, Fm and Fm′ in accordance to the following standard equations: Fv/Fm = (Fm − F0)/Fm, Ф_PSII_ = (Fm′ − F)/Fm, NPQ = (Fm − Fm′)/Fm′. The leaf area was measured using the software of Plant Explorer^Pro+^ [44,45].

### 4.4. Determining Transpiration and the Water Content 

The relative transpiration was estimated using the crop water stress index (CWSI) and was determined using a thermal imager Testo 885 (Testo, Lenzkirch, Germany). Each group of plants was photographed simultaneously with reference samples of absolutely wet and dry standards. The obtained images with temperature values were processed using IRSoft software. On the thermogram of each experimental group, 20 independent points on the leaves, as well as on the reference samples, were noted. The CWSI was calculated by the following equation [46,47]:

CWSI = (T_dry_ − T)/(T_dry_ − T_wet_).

The relative water content (RWC) in the plant tissues and soil was calculated using the ratio of fresh weight (FW) and dry weight (DW) of the leaves. The relative water content was calculated by the following equation:

RWC (%) = 100(FW − DW)/FW.

### 4.5. Statistics

Three independent experiments were performed. In each of them, the experimental groups were represented by at least 6 repetitions. The mean value and the standard error of the mean were calculated using MS Excel 2016. The reliability of the differences between the groups was assessed by Student’s *t*-test.

## Data Availability

The data presented in this study are available on request from the corresponding author.
